# Impact of changing guidelines on genetic testing and surveillance recommendations in a contemporary cohort of breast cancer survivors with family history of pancreatic cancer

**DOI:** 10.1038/s41598-021-91971-0

**Published:** 2021-06-14

**Authors:** Annie Wang, Jessica N. Everett, Jennifer Chun, Cindy Cen, Diane M. Simeone, Freya Schnabel

**Affiliations:** 1 Department of Surgery, New York University Langone Health, New York, NY USA; 2 Department of Medicine, New York University Langone Health, New York, NY USA; 3 Department of Pathology, New York University Langone Health, New York, NY USA; 4 Perlmutter Cancer Center, New York University Langone Health, 160 East 34th St., New York, NY 10016 USA

**Keywords:** Breast cancer, Cancer genetics, Pancreatic cancer

## Abstract

Changing practice guidelines and recommendations have important implications for cancer survivors. This study investigated genetic testing patterns and outcomes and reported family history of pancreatic cancer (FHPC) in a large registry population of breast cancer (BC) patients. Variables including clinical and demographic characteristics, FHPC in a first or second-degree relative, and genetic testing outcomes were analyzed for BC patients diagnosed between 2010 and 2018 in the NYU Langone Health Breast Cancer Database. Among 3334 BC patients, 232 (7%) had a positive FHPC. BC patients with FHPC were 1.68 times more likely to have undergone genetic testing (p < 0.001), but 33% had testing for *BRCA1/2* only and 44% had no genetic testing. Pathogenic germline variants (PGV) were identified in 15/129 (11.6%) BC patients with FHPC, and in 145/1315 (11.0%) BC patients without FHPC. Across both groups, updates in genetic testing criteria and recommendations could impact up to 80% of this cohort. Within a contemporary cohort of BC patients, 7% had a positive FHPC. The majority of these patients (56%) had no genetic testing, or incomplete testing by current standards, suggesting under-diagnosis of PC risk. This study supports recommendations for survivorship care that incorporate ongoing genetic risk assessment and counseling.

## Introduction

Breast cancer (BC) is the most common cancer in women, with a 5-year survival rate of > 90%. More than 3.8 million women in the United States are BC survivors, representing four in ten of all female cancer survivors. As survival has steadily improved, more attention has shifted to BC survivors’ post-treatment needs, including addressing risk and surveillance for second primary malignancies^1^.

In contrast, pancreatic cancer (PC) is the 3rd leading cause of cancer-related death in the United States, with a dismal 5-year survival rate of 10%. In 2020, approximately 57,600 men and women will be diagnosed with PC and more than 47,050 will die from the disease^2^. By the time symptoms of PC develop, the cancer is typically advanced and not amenable to surgical resection. For the 15% of patients presenting with localized disease that can be surgically treated, the 5-year survival rate approaches 60–70% for tumors less than 1 cm in size^3^, highlighting the potential value of early detection. Current United States Preventive Services Task Force (USPSTF) recommendations do not support screening for PC in the general population^4^, but guidelines for screening with MRI/MRCP and endoscopic ultrasound in a defined population at high risk for PC due to family history and/or genetic risk factors exist^5,6^. Emerging data suggests a benefit in PC screening in this high-risk population, including improved identification of early stage PC, and improved 3-year survival^7–9^. Identifying patients who meet criteria for PC surveillance based on family history and gene status is an important goal for early detection.

Several lines of evidence support a link between BC and PC risk in some families. A cohort analysis of 5799 families with multiple or early onset cases of breast or ovarian cancer demonstrated increased risk of PC in families with pathogenic germline variants (PGVs) in *BRCA1* or *BRCA2*^10^*.* The study also found elevated PC risk in families negative for *BRCA1/2,* suggesting that elevated PC risk in BC families was not fully explained by *BRCA* PGV^10^. Germline testing identifies a clinically relevant PGV in up to 10% of patients with PC^11–15^, and one study found that PGVs were more common in PC patients with a history of BC in themselves or a first degree relative^14^. A case–control study found evidence for an independent association of PC risk with PGVs in six genes: *ATM*, *BRCA1*, *BRCA2*, *CDKN2A*, *MLH1*, and *TP53*^16^, five of which (excluding *CDKN2A*) have a clear link to breast cancer risk^17–20^. PGVs in *PALB2* and *STK11* have also been linked to increased risk for both PC and BC^21–23^. Current NCCN clinical practice guidelines recommend consideration of comprehensive testing with a multi-gene panel for BC patients meeting specific testing criteria based on age, hormone receptor status, Ashkenazi Jewish ancestry, and family history criteria, as well as for all PC patients. The guidelines also recommend consideration of multi-gene panel testing for patients meeting criteria who previously tested negative with limited testing (e.g. single gene analysis)^24^.

The evolving knowledge about underlying PGVs linking BC and PC risk, the utility of multi-gene panel testing, and the emergence of data supporting the benefit of PC screening in high-risk individuals have potential implications for long term care of BC survivors. The purpose of this study was to investigate genetic testing patterns and outcomes, along with reported family history of PC, in a registry population of patients with BC. We sought to better characterize the implications of changing practice guidelines and clinical recommendations for BC survivors, utilizing the specific example of PC risk.

## Methods

All newly diagnosed BC patients undergoing definitive surgery enrolled in the NYU Langone Perlmutter Cancer Center Breast Cancer Database between January 2010 and December 2018 were included in the study. Variables of interest included patient demographics (age at BC diagnosis, race), family history of PC in a first or second degree relative, genetic testing type (*BRCA1*/*2* only or multi-gene panel testing defined as any test including additional genes beyond *BRCA1* and *BRCA2*), tumor characteristics, and BC recurrence. All clinical data were obtained from electronic medical record review and detailed questionnaires completed by participating patients. Details of the Breast Cancer Database have been previously described^25^. This study was approved by the NYU Institutional Review Board, and research was performed in accordance with relevant guidelines/regulations. Patients signed informed consent prior to database enrollment.

Descriptive analyses were used to summarize the data distribution of variables between the patients who had family history of PC compared to those who did not have family history of PC. Statistical analyses included Pearson’s chi-square and Wilcoxon rank-sum with a significance level of α = 0.05. All analyses were done using SAS software, version 9.4 (SAS Institute).

## Results

A total of 3334 BC patients enrolled in the Breast Cancer Database during the 8 year study period. Patient demographics and clinical characteristics are shown in Table 1. Among the total BC patient population, 232 (7%) reported a family history of PC in a first or second degree relative. Breast cancer patients with a family history of PC were 1.93 times more likely to be of white race (p < 0.001) and 1.68 times more likely to have had genetic testing (56% vs 44%, p < 0.001) than those without family history of PC. There were no other significant differences in demographic or clinical variables between patients with and without family history of PC (Table 1). In the 129 patients with FHPC who underwent genetic testing, 15 (11.6%) had PGVs identified, with 9/15 (60%) occurring in genes with a clear link to PC risk. Among BC patients without FHPC, 145/1315 (11.0%) of patients had a PGV identified, with 123/145 (85%) occurring in genes linked to PC risk (Table 2). The majority of BC patients who had genetic testing, with and without family history of PC, had testing for *BRCA1* and *BRCA2* only (58.9% and 63% respectively) rather than a multi-gene panel.

**Table 1:** Demographic and clinical characteristics.

	FHPC positive N = 232	FHPC negative N = 3102
**Median age at diagnosis**	59.4	59.7
Range	(25.2–95.7)	(22.5–95.1)
**Gender**		
Male	1 (0.4)	20 (0.6)
Female	231 (99.6)	3082 (99.4)
**Race and ethnicity**		
White	196 (84.9)	2289 (73.8)*
Black	14 (6.1)	284 (9.2)
Asian	12 (5.2)	317 (10.2)
Other	1 (0.4)	18 (0.6)
Hispanic	8 (3.4)	193 (6.2)
Ashkenazi Jewish	75 (32.3)	872 (28.1)
**Tumor stage**		
0	49 (21.1)	645 (20.8)
I	122 (52.6)	1550 (50.0)
II	51 (22.0)	680 (21.9)
III	10 (4.3)	207 (6.7)
IV	0	17 (0.6)
**Histology**		
DCIS	49 (21.1)	646 (20.8)
Invasive ductal	147 (63.4)	2015 (65.0)
Invasive lobular	29 (12.5)	292 (9.4)
Other invasive	7 (3.0)	149 (4.8)
**Mean size (cm)**	1.63	1.70
Range	0.0–8.0	0.0–12.5
**Invasive grade**		
Grade 1	24 (13.7)	343 (14.3)
Grade 2	99 (56.6)	1285 (53.7)
Grade 3	52 (29.7)	764 (32.0)
**Hormone receptor status**		
ER positive	197 (84.9)	2603 (84.5)
PR positive	173 (74.6)	2223 (72.2)
**HER2/neu status**		
Positive	19 (10.7)	330 (13.7)
Negative	153 (86.0)	2012 (83.3)
Equivocal	6 (3.3)	73 (3.0)
Recurrence	10 (4.3)	129 (4.2)

*FHPC* family history of pancreatic cancer.

*p = 0.005.

**Table 2:** Genetic testing outcomes in BC patients.

	FHPC positive (n = 232)	FHPC negative (n = 3102)
**Genetic testing**		
Yes	129 (55.6)	1315 (42.7)*
No	103 (44.4)	1769 (57.3)
Unknown	–	18 (0.6)
**Test type**		
*BRCA1/2*	76 (58.9)	828 (63.0)
Multi-gene panel	53 (41.1)	488 (37.0)
**PGV identified—total**	15 (11.6)	145 (11.0)
**ATM**	**–**	**5**
**BRCA1**	**2**	**55**
**BRCA2**	**6**	**53**
**CDKN2A**	**–**	**3**
**MLH1**	**–**	**2**
**MSH6**	**–**	**1**
**PALB2**	**1**	**3**
**TP53**	**–**	**1**
*APC* (I1307K)	3	1
*BARD1*	–	2
*BRIP1*	–	1
*CDH1*	–	1
*CHEK2*	1	10
*MUTYH*	1	4
*NBN*	–	2
*PTEN*	–	1
*RAD51C*	1	–

Bold area represents genes with PC screening recommendations.

*p < 0.001.

## Discussion

In our BC study population of over 3000 patients, 7% of BC patients reported a family history of PC in a first or second degree relative. Genetic testing was completed in 56% of BC patients with FHPC, and 11.6% carried a PGV with clinical relevance. Genes linked to PC risk represented 60% of the findings (*BRCA1*, *BRCA2*, and *PALB2*), and these patients meet current published guidelines recommending PC surveillance with annual imaging beginning at age 50 or 10 years younger than the earliest diagnosis of PC in the family^5,6^. Importantly, several BC patients without a family history of PC at the time of diagnosis also carried PGVs in genes linked to PC risk (*ATM*, *BRCA1, BRCA2, CDKN2A*, *PALB2*, and *MLH1*). All of these patients would benefit from follow up care that includes updating family history for new PC diagnoses that could impact care, as well as receiving information about changes in PC surveillance recommendations over time.

Our study found that changes in criteria for genetic testing, including expanded age range and added family history components, and the addition of multi-gene panel testing as a recommended approach have potential impact on a majority of BC patients. A substantial proportion of BC patients in the study population, 43% overall, had some type of genetic testing. This proportion is higher than was found in a recent population based analysis of BC patients^26^, and could reflect the demographic characteristics of our study population which was 28% Ashkenazi Jewish, or differences in utilization at an academic hospital. Despite relatively high test utilization, 44% of BC patients with a family history of PC and 57% of those without family history of PC had no genetic testing of any kind. This lack of testing could be attributed to a variety of reasons, including not being referred for testing by treating physician^27–29^, or declining testing for reasons including cost^28,29^ or anxiety about potential second primary cancers and impact on future generations^30^. Another possibility is that patients did not meet testing criteria at the time of the initial diagnosis. NCCN criteria for genetic testing and screening have evolved and expanded over time, and BC survivors could benefit from periodic re-assessment that considers current criteria along with any changes in the family history of cancer. Additionally, the performance of genetic testing guidelines, including NCCN and Medicare guidelines, have come into question with similar PGV detection rates in BC patients who met criteria compared to those who did not^31,32^. One study found that the sensitivity of NCCN criteria for genetic testing were improved from 70 to 90% by including all women with breast cancer diagnosed under age 65^33^. Based on this data, the American College of Breast Surgeons put forth a statement in 2019 advocating for offering genetic testing to any BC patient, regardless of age or family history^34^. This statement would have relevance for the 56% of BC patients in our study who had no testing at all.

Among those in our study who completed genetic testing, 62.6% (41% of those with family history of PC) had testing only for *BRCA1* and *BRCA2*. This could reflect differences in availability and utilization of testing for patients seen 2014–2018 when multi-gene panel testing became more broadly available. This change was brought about by evolution in testing technology decreasing sequencing cost, along with a June, 2013 United States Supreme Court decision invalidating patents on the sequence of *BRCA1* and *BRCA2*^35,36^. While we did not have sufficient data available on exact dates of testing to complete a specific analysis in our population, one study of test utilization in BC patients showed a shift from 74% *BRCA1/2* only in 2013 to 33.5% *BRCA1/2* only by 2015^37^. NCCN criteria as recently as 2016 continued to recommend specific syndrome testing as the preferred approach, with consideration of multi-gene panel testing only for patients negative for single syndrome testing and with a family history suggestive of inherited cancer risk. Clinical caution in adopting multi-gene panel tests centered primarily on concerns about variants of uncertain significance, and lack of well-characterized risk information and management strategies for several of the low or moderate risk genes included in panels^38,39^. Additionally, questions about cost effectiveness^40^ have been addressed over time^41,42^ allowing insurers to adjust coverage criteria. Multiple studies have now documented additional relevant PGV findings in women with breast cancer offered multi-gene panel testing^43–46^. As in our population, the most common genes beyond *BRCA1*/*2* identified on multi-gene panels included several with links to PC risk. Current NCCN guidelines have shifted to recommend consideration of multi-gene panel testing both as a first line test, and for patients previously negative for *BRCA1*/*2*^24^. Genetics providers have attempted re-contact of patients via mail to notify them of updated testing, but subsequent test uptake has been very low^47,48^. Integrating genetic services into cancer survivorship care could help to bridge this gap, and has shown efficacy in a pediatric survivor clinic setting^49^.

The American Cancer Society and American Society of Clinical Oncology have developed guidelines for survivorship care that address multiple relevant issues for BC survivors including risk evaluation and genetic counseling, as well as screening for second cancers^1^. These guidelines address the possibility that genetic counseling and genetic testing may not have been offered to a patient meeting criteria, or that family history may have changed leading to a patient meeting criteria who did not at initial diagnosis. In addition, other factors may change, including identification of new susceptibility genes or advances in testing technology, updates to genetic testing criteria, or changes in cancer screening recommendations for individuals with relevant PGVs or family history risk factors^50^, suggesting the importance of ongoing repeated genetic risk assessment in cancer survivors^50^. Our findings provide support for this recommendation.

The study has some limitations, including reliance on retrospective analysis of patient-reported family history, which may be incomplete, or may have changed to include new cancer diagnoses since the time of last data collection. This could result in misclassification of BC patients with and without family history of PC. For the purposes of data entry, “multi-gene panels” included testing for any gene(s) beyond *BRCA1/2*, and specific information about which genes and total number of genes analyzed was not documented. This could lead to under-identifying the number of patients who could benefit from updated testing. Patients within the NYU Langone Health system may have had later genetic testing at a different institution or with an outside provider that was not shared with their NYU provider and thus not available for our record review. Finally, variants of uncertain significance were not included as part of this data review.

## Conclusions

In this study of a large cohort of BC patients who underwent surgical resection, we found that seven percent of BC patients had a positive family history of PC. Of these, 44% did not have genetic testing of any kind and another 33% had testing for only *BRCA1*/*BRCA2* germline mutations, rather than multi-gene panel testing. Based on this data, updates in genetic testing criteria and recommendations for multi-gene panel testing have potential impact on up to 80% of BC survivors. Given the number of known genes associated with risk for both BC and PC, and published recommendations for pancreatic surveillance in carriers of PGV with a family history of PC, patients with BC could benefit from ongoing care to re-evaluate genetic risk and provide updated screening recommendations. This study supports recommendations for survivorship care that incorporates ongoing genetic risk assessment and counseling.

## Data Availability

The datasets generated during and/or analyzed during the current study are available from the corresponding author on reasonable request.
